# Improving rehabilitation in sarcopenia: a randomized‐controlled trial utilizing a muscle‐targeted food for special medical purposes

**DOI:** 10.1002/jcsm.12532

**Published:** 2020-09-22

**Authors:** Mariangela Rondanelli, Emanuele Cereda, Catherine Klersy, Milena Anna Faliva, Gabriella Peroni, Mara Nichetti, Clara Gasparri, Giancarlo Iannello, Daniele Spadaccini, Vittoria Infantino, Riccardo Caccialanza, Simone Perna

**Affiliations:** ^1^ IRCCS Mondino Foundation Pavia Italy; ^2^ Department of Public Health, Experimental and Forensic Medicine, Unit of Human and Clinical Nutrition University of Pavia Pavia Italy; ^3^ Clinical Nutrition and Dietetics Unit Fondazione IRCCS Policlinico San Matteo Pavia Italy; ^4^ Biometry and Clinical Epidemiology Service Fondazione IRCCS Policlinico San Matteo Pavia Italy; ^5^ Azienda di Servizi alla Persona (ASP) di Pavia University of Pavia Pavia Italy; ^6^ Directorate General, Azienda di Servizi alla Persona (ASP) di Pavia Pavia Italy; ^7^ Department of Biology, College of Science University of Bahrain Kingdom of Bahrain

**Keywords:** Leucine, Nutritional support, Rehabilitation, Sarcopenia, rehabilitation, Vitamin D, Whey protein

## Abstract

**Background:**

Sarcopenia is a disease associated with aging and a negative prognosis. Consensus‐based treatment consists in targeting muscle mass and function through physical exercise, optimization of protein intake, and vitamin D supplementation, but evidence is lacking. We evaluated the safety and efficacy of a muscle‐targeted nutritional support on the outcome of a physical exercise rehabilitation programme.

**Methods:**

In a single‐site, double‐blind, randomized, controlled trial (NCT03120026; May 2017 to December 2018), old (≥65 years) adults [*N* = 140 (63% female patients; age, 81 ± 6 years)] without severe cognitive impairment, who were found to have sarcopenia by European Working Group on Sarcopenia in Older People 2010 criteria and hospitalized for physical rehabilitation, were randomized to receive until discharge (for at least 4 weeks and up to 8 weeks) a whey protein‐based nutritional formula enriched with leucine and vitamin D or an iso‐caloric control formula twice daily in addition to a standard hospital diet. The primary endpoint was the change in 4 m gait speed per month. Key secondary endpoints addressed the change in physical performance: chair‐stand test, timed up and go test, and short physical performance battery. Other secondary outcomes were the change in functional status, muscle strength and mass, cognitive status, and quality of life. The proportion of patients who improved their rehabilitation intensity profile and overall economic benefits (using length of stay and duration of rehabilitation as surrogate measures) were also evaluated.

**Results:**

A total of 161 patients were screened and 140 were randomized to study interventions. Thirteen patients (experimental, *n* = 6; placebo, *n* = 7) discontinued the intervention because they disliked the product and intention‐to‐treat analyses were based on patients reassessed at discharge [*n* = 127 (66% female patients; age, 81 ± 6 years)]. Supplementation with the experimental formula (*n* = 64) resulted in greater increase in mean gait speed {0.061 m/s/month [95% confidence interval (CI), 0.043 to 0.080]} than placebo [*n* = 63; −0.001 m/s/month (95%CI, −0.008 to 0.006)]: mean difference, 0.063 m/s/month (95%CI, 0.043 to 0.082) (*P* < 0.001). A significant effect was also found for muscle mass (*P* < 0.03) and all key secondary outcomes, functional and cognitive endpoints (*P* < 0.001 for all). Supplementation resulted also in higher proportion of patients improving their rehabilitation intensity profile (*P* = 0.003) and being discharged home (*P* = 0.002); shorter rehabilitation (*P* < 0.001); and hospital stay (*P* < 0.001).

**Conclusions:**

In old adults with sarcopenia admitted to hospital for rehabilitation the consumption of a whey protein‐based nutritional formula enriched with leucine and vitamin D improved physical performance and function, as well as muscle mass, and reduced the intensity and costs of care.

## 1 Introduction

Age‐related and disease‐related loss of muscle mass and strength induces important negative pathophysiological changes in the elderly, such as reduction in the ability to maintain balance, changes in glucose homeostasis, thermoregulation and bone nutrition, and reduced basal metabolic rate/energy production.^1^, ^2^, ^3^, ^4^ These consequences contribute considerably to the progression of loss of self‐sufficiency on account of the reduction in both dynamic and static muscle strength, increase in morbidity, and susceptibility to multiple disorders; overall, they contribute to a general condition called *sarcopenia*.^4^ It is noteworthy that, although several common geriatric conditions (malnutrition, frailty, cachexia, and sarcopenia) overlap to a considerable extent,^5^ an independent International Classification of Disease‐10 has been recently assigned to sarcopenia.^6^

A number of factors may contribute to the onset of sarcopenia. Sudden and important muscle mass loss is a common occurrence following a period of immobility or reduction in mobility, such as admission to hospital, and only 3 days of bed rest can result in the loss of >1 kg of muscle mass in elderly patients.^7^ Nonetheless, sarcopenia is a disease that frequently occurs in the community, where a sedentary lifestyle and inadequate dietary intake of proteins and specific micronutrients (e.g. vitamin D) can trigger and accelerate its progression.^2^, ^8^, ^9^

Taking into account the time trends in life expectancy, the treatment of this disease is a key issue with important pharmaco‐economic implications.^10^ Interventions should modulate anabolic and catabolic pathways within the muscle but, despite a substantial increase in the understanding of the molecular basis, approved agents are limited and not free of side effects.^11^ At present, the basic treatment of sarcopenia consists in targeting muscle mass and function through physical exercise (resistance training), optimization of protein intake, and vitamin D supplementation.^8^, ^9^, ^11^

In respect to nutritional intervention, particularly to optimize protein intake in advanced age, attention should be paid not only to the amount of proteins provided but also to their quality.^12^, ^13^ Whey proteins have proved to enable greater anabolic stimulation due to their faster digestion—resulting in more rapid increase in plasma amino acid levels—and high content in essential amino acids.^13^ Among essential amino acids, leucine has been found to stimulate anabolism independently.^14^ It has been recommended that a per‐meal anabolic threshold of essential amino acids, particularly in terms of leucine (2.5–2.8 g/meal), should be achieved at least twice daily.^15^ Besides, vitamin D supplementation was found to result in improved muscle strength, particularly in old adults with low serum levels of the vitamin^16^ and to promote muscle anabolism also through a positive interaction among all these nutrients.^17^

Recent studies have demonstrated that the use of a muscle‐targeted food for special medical purposes (a mixture of whey proteins enriched with essential amino acids, especially leucine, vitamin D, and calcium)^18^, ^19^, ^20^, ^21^ improves muscle mass and strength, as well as physical performance regardless of sarcopenia.^21^ However, although clinical trials in sarcopenia should address the recovery of physical functioning,^9^, ^22^ the efficacy of the muscle‐targeted food for special medical purposes on physical performance has never been evaluated in combination with physical exercise in a high‐quality trial. Furthermore, there are no data on the economic benefits deriving from nutritional support in in‐patient rehabilitation facilities.

The purpose of this study was to assess the efficacy of a muscle‐targeted nutritional supplementation on physical performance, functional, and muscle mass recovery in older sarcopenic patients admitted to an in‐patient rehabilitation facility, as well as to gather information on its economic benefits.

## 2 Methods

### 2.1 Standard protocol approvals, registrations, and patient consents

This study was conducted in accordance with good clinical practice and with the ethical standards laid down in the 1964 Declaration of Helsinki and its later amendments. The study protocol was approved by the Institutional Ethics Committee and written informed consent was obtained from every patient entering the pre‐treatment phase. The study was registered with ClinicalTrials.gov (NCT03120026) (Data S1).

### 2.2 Study design

This was a single‐site (Geriatric Physical Medicine and Rehabilitation Division, Santa Margherita Hospital, Azienda Human Service of Pavia), randomized (1:1), parallel‐group, double‐blind, controlled, 8 week clinical trial (May 2017 to December 2018). Patients were assessed at admission and discharge after minimum 4 weeks and maximum 8 weeks of intervention with physical exercise and nutritional supplementation or an iso‐caloric control formula. Allocation to the intervention groups occurred via a computer‐generated random blocks randomization list (varying block sizes). Random assignments were concealed in sealed envelopes.

### 2.3 Participants

Participants were old adults (age ≥ 65 years) candidates for in‐patient rehabilitation without severe cognitive impairment [Mini Mental State Examination (MMSE) ≥18],^23^ who were found to have sarcopenia as defined according to European Working Group on Sarcopenia in Older People (EWGSOP) 2010 criteria^8^ in terms of the outcome of body composition by bioimpedance analysis [(skeletal muscle mass/body weight × 100) ≤ 37% in men and ≤ 28% in women], handgrip strength, and gait speed. Accordingly, subject with low muscle mass and low muscle strength or physical performance was included. We excluded subjects who had severe renal failure (glomerular filtration rate < 30 mL/min), moderate to severe liver failure (Child‐Pugh class B or C), endocrine diseases associated with calcium metabolism disorders (except osteoporosis), known psychiatric disorders, cancer (over the past 5 years), or hypersensitivity to any component of the investigational nutritional supplement and those who were adhering to a high‐energy or high‐protein diet (up to 3 months before starting the study) or were taking calcium supplements (exceeding 500 mg daily) or vitamin D supplements [exceeding 10 μ daily (400 IU) daily] or protein/amino acid supplements. Patients unable to take oral therapy and those receiving or with indication for artificial nutrition or who had been included in another clinical nutrition trial were also excluded. Investigator's uncertainty about the willingness or ability of the subject to comply with the protocol requirements was also considered as an additional exclusion criterion.

### 2.4 Nutritional interventions

An individualized dietary programme was drawn up for each patient, taking nutritional and mastication issues, as well as any swallowing issues into consideration. In addition to hospital diet, subjects were randomly allocated to receive twice daily:

- Experimental formula: A whey protein‐based food for special medical purposes enriched with leucine and vitamin D (Fortifit®, Nutricia). Each serving consisted of 40 g of powder (vanilla or strawberry flavor), providing 150 kcal and containing 20 g of whey proteins, 2.8 g of leucine, 9 g of carbohydrates, 3 g of fat, 800 IU of vitamin D, and a mixture of vitamins, minerals (calcium, 500 mg), and fibres.^18^, ^19^, ^20^, ^21^
- Control formula: An isocaloric formula consisting of 40 g of a flavoured (vanilla or strawberry) powder containing maltodextrins.

The intervention formula was reconstituted with 100–150 mL water and administered at breakfast and in the afternoon. In the event of dysphagia to liquids, the density of the reconstituted formula was increased, as appropriate. The actual experimental and control formula were given in identical containers devoid of any labelling for at least 4 weeks (minimum duration of the rehabilitation) up to 8 weeks (maximum duration of the rehabilitation). Compliance with intake of nutritional interventions was monitored by entering the number of servings consumed every day in a diary.

### 2.5 Physical intervention

An individualized, moderate‐level (Borg Rate of Perceived Exertion scale score of 12–14^24^) physical fitness and muscle mass promoting program was set up for all in‐patients. Trained staff supervised all exercise sessions, monitoring the individual exercise ability of each patient and adjusting the intensity level, as appropriate. The intervention consisted of exercise sessions daily, five times per week. The initial duration of each session was 20 min, and it could be increased progressively, along with the intensity of the exercises, up to 30 min. All sessions included the following:

- 5 min warm‐up
- 5 to 10 min progressive sequence from seated to standing muscle‐strengthening exercises: toe raises, heel raises, knee lifts, knee extensions in the seated position; hip flexions and lateral leg raises standing next to a chair used for stability; ankle‐weight bearing exercises (seated knee flexion and extension, standing knee flexion and extension), with weights ranging from 0.5 to 1.5 kg as appropriate (in accordance with each participant's strength as the resistance progressively increased); leg extensions and hip flexions using resistance bands. Upper‐body exercises were also performed and included double‐arm pull downs and biceps curls. Patients were asked to perform up to eight repetitions, as appropriate
- 5 to 10 min balance and gait exercises: one‐leg stands, tandem stands, multidirectional weight shifts, tandem walk, as well as practicing proper gait mechanics focusing on balance maintenance and increasing stride length, while changing direction and/or gait pattern
- 5 min cool‐down.

The minimum duration of the physical intervention program was 4 weeks, and it could be prolonged up to 8 weeks according to the results obtained. Specifically, the decision to finish the rehabilitation and to discharge the patient was taken by a multidisciplinary team (geriatrician, physiatrist, physiotherapist, and nurse) once the duration of each exercise session was stabilized to 30 min and no increase in intensity could be considered for five consecutive days.

### 2.6 Assessments

In addition to demographic (age and gender) and general medical history (main admittance diagnosis, number of comorbidities, and medications) data collection, the following assessments were carried out:

- Nutritional assessment: Body weight (to the nearest 0.1 kg) and height (to the nearest 0.5 cm) were measured according to standard procedures, and body mass index (BMI) was derived accordingly.^25^ A trained dietitian was responsible for the evaluation of calorie and protein intake. At study inclusion a 24 h dietary recall (with the aid of the caregiver) was performed with the help of a food atlas,^26^ while at the end of study a calibrated dietetic spring scale was used to weigh all foods served and returned on consecutive days. A computer program (DR3 v3.1.0; Sintesi Informatica Srl, Milano, Italy) was used to estimate the energy and the macronutrient content of food consumed, including nutritional supplementation. Finally, nutritional status was rated by means of the Mini Nutritional Assessment (MNA®), a brief questionnaire based on an anthropometric assessment (BMI and weight loss), a general assessment (lifestyle, medication, and mobility), and a dietary assessment (number of meals, food and fluid intake, self‐assessment of autonomy of eating, and self‐perception of health and nutrition).^27^
- Body composition assessment: In the screening phase, the presence of low skeletal muscle mass was diagnosed [(skeletal muscle mass/body weight × 100) ≤37% in men and ≤ 28% in women] using bioelectric impedance analysis 101 (Akern s.r.l., Florence, Italy).^8^, ^28^ Then, appendicular muscle mass (AMM) and total body skeletal muscle mass (for the calculation of skeletal muscle mass index [SMMI]) were evaluated using dual‐energy X‐ray absorptiometry (Lunar Prodigy, GE Medical Systems).
- Evaluation of physical performance: It comprised multiple tests. Gait speed was evaluated by the 4 m walking test, asking the patients to walk at their usual pace and taking into account the best time of two attempts. Patients could use an assistive device, if needed. Specifically, the patient was asked to walk down a hallway through a 1 m zone for acceleration, a central 4 m “testing” zone, and a 1 m zone for deceleration (the patient should not start to slow down before the 4 m mark), starting and stopping the timer with the first footfall after the 0 m line and the 4 m line, respectively.^29^ Lower body leg strength and endurance were investigated through the chair‐stand test (time required to rise five consecutive times from a chair without arm rests).^30^ Composite evaluation of mobility, balance, walking ability, and fall risk was performed using the timed up and go (TUG) test, which assesses the time taken to rise from an arm chair, walk 3 m, turn, walk back, and sit down again.^31^ Finally, we considered the Short Physical Performance Battery (SPPB) which consists of three components: gait speed, chair‐stand test, TUG, and balance (three different tests assessing ability to stand with the feet together in the side‐by‐side, semi‐tandem, and tandem positions). Accordingly, each component was scored from 0 (*not possible*) to 4 (*best performance*); the scores add up to a total score ranging from 0 to 12.^32^
- Functional status evaluation: It included muscle strength measured as handgrip strength [according to standard procedures by a hydraulic hand dynamometer (Jamar 5030 J1; Sammons Preston Rolyan; Bolingbrook, Canada; accuracy 0.6 N)], the Barthel Index [covering all the aspects of self‐care independence in daily living activities, including transfer, walking, stairs, toilet use, dressing, feeding, bladder, bowel, grooming, and bathing; score range, 0 (*completely dependent*)–100 (*complete self‐sufficiency*)],^33^ activities of daily living (ADL) score,^24^ and the Tinetti scale that measures characteristics associated with falls, assessing balance (14 items; 24 points), and gait (10 items; 16 points) for a total score up to 40 (the higher the score, the better the performance).^35^
- Evaluation of cognitive functions: It included the MMSE (a 30‐point questionnaire used to measure cognitive impairment assessing functions including registration, attention and calculation, recall, language, ability to follow simple commands, and orientation)^23^ and the Trail making test, a neuropsychological test of visual attention and task switching, providing information about visual search speed, scanning, speed of processing, mental flexibility, as well as executive functioning (the score is obtained as the number of seconds needed to complete the test)^36^
- Quality of life (QoL)assessment: Participants were tested with the Short‐Form 12‐Item Health Survey (SF‐12), a short, generic health‐status measure reproducing the 2 summary scores of the SF‐36—the physical component summary score and the mental component summary score—by addressing eight health domains (physical functioning, role physical, bodily pain, general health, vitality, social functioning, role emotional, and mental health).^37^
- Biochemical assessment: Venous blood samples were drawn after an overnight fast and used for the evaluation of routine parameters (total blood count, glucose, transaminases, albumin, creatinine, blood urea nitrogen, serum electrolytes, transferrin, and total cholesterol), as well as C‐reactive protein and 25‐hydroxyvitamin D (25(OH)D) levels.
- Assessment of complexity of assistance needs: The rehabilitation profile system was used to rate the complexity of assistance needed by the patient.^38^ Profiles are defined according to the assessment of four kinds of intervention and their interaction (general assistance, functional reactivation and recovery, medical support, and social support). Patients entering an intermediate rehabilitation programme as in‐patients can be assigned any one of five different profiles:
  1. Profile 1: low assistance and medical needs; patient requires mainly general assistance;
  2. Profile 2: intermediate general assistance needs, but low medical needs; patient requires mainly general assistance, as well as functional reactivation and recovery;
  3. Profile 3: high general assistance needs and intermediate medical needs; patient requires mainly general assistance, functional reactivation and recovery, as well as intermediate medical support;
  4. Profile 4: high assistance and medical needs; patient requires general assistance, functional reactivation and recovery, as well as medical support on account of important concomitant diseases;
  5. Profile 4B DEMENTIA: high assistance and medical needs. Patient suffers from dementia and therefore requires a lot of general assistance, functional reactivation and recovery, as well as medical and social support provided by highly trained professionals.

### 2.7 Efficacy endpoints

The primary efficacy end‐point was the mean change (between admission and discharge) in gait speed per month (m/s/month). The key secondary endpoints were change (per month) in physical performance outcome measures: chair‐stand test, TUG, SPPB. Other secondary outcome variables were changes (per month) in: handgrip strength, Tinetti scale, Barthel Index, ADL, body weight, AMM, SMMI, cognitive status (Trail making test and MMSE), and QoL. The proportion of patients who improved their rehabilitation intensity profile, the modality of discharge (home vs. institution—with institutionalization indicated if SPPB ≤5 and/or Barthel Index <45 and/or Tinetti scale <18) and overall economic benefits [using length of stay (LOS; days) and total duration of rehabilitation (minutes) as surrogate measures] were also evaluated. Finally, nutritional exploratory efficacy end‐points were the changes in protein and energy intake, MNA score, C‐reactive protein, vitamin D, total cholesterol, albumin, and creatinine.

### 2.8 Adverse events

Patients were actively monitored for the occurrence of any potential gastrointestinal side effect associated with the consumption of the nutritional intervention formula (common adverse events). The occurrence of any unexpected serious adverse event was also recorded.

### 2.9 Statistical analysis

In the absence of preliminary data to estimate the expected treatment difference, the sample size was set at 128 patients reaching the evaluation of the primary endpoint (64 per arm) to achieve a statistical power of 80% (type I error 5% using a two‐tailed test) to detect a clinically meaningful difference [mean treatment difference/standard deviation (effect size) = 0.5].^39^, ^40^ Allowing for a 10% drop‐out rate in each arm, it was decided to randomize 140 patients (70 per treatment arm).

Expecting a treatment effect also on the LOS, changes over time in both primary and secondary continuous outcome variables were normalized by the duration of observation in months. Changes were calculated so that an improvement would result in a positive value in favour of the experimental formula (as either final—initial value or initial—final values).

The efficacy analysis population included the patients reaching the primary endpoint evaluation and it was performed according to the intention to treat (ITT) principle (modified ITT population).

The change in gait speed was compared between groups with a generalized linear regression model using Huber–White robust standard errors to account for variance inhomogeneity. Then a series of supportive analyses of the primary endpoint were performed. First, we conducted a multivariable model, to adjust for potential confounders regardless of differences in baseline features, including gender, age, and monthly change in energy intake, in creatinine, and in total cholesterol. Second, a conservative sensitivity analysis of the primary endpoint using the worst possible outcome of the study for patients dropping out was performed.

Group comparison was performed for all secondary endpoints on a continuous scale using an unadjusted generalized linear regression model, as described above. For both primary and secondary outcome variables the mean change within groups was also assessed. For secondary endpoints on a binomial scale, a generalized linear model for the binomial family was used. For all endpoints the treatment effect (mean or frequency difference) and 95% CI were reported.

All patients consuming at least one serving of the nutritional formula were included in safety analysis.

Continuous variables were described as mean and standard deviation (SD) or median and 25th to 75th percentiles according to normality of distribution. Categorical variables were reported with counts and percent. All analyses were performed with Stata 15.1 (StataCorp, College Station, TX, USA). The level of significance was set at the two‐tailed *P* value < 0.05.

### 2.10 Funding and role of the sponsor

This work was supported and sponsored by the Azienda di Servizi alla Persona of Pavia and the University of Pavia. The sponsor had no role in the design and conduct of the study, in the collection, management, analysis, and interpretation of the data or in the preparation, review, or approval of the manuscript.

## 3 Results

**Figure 1:**
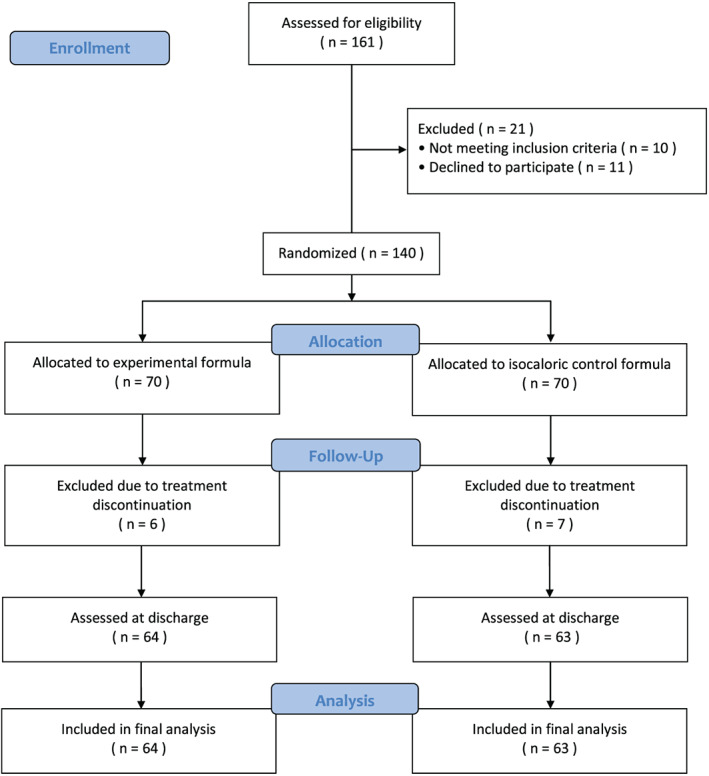
Study flow diagram

A total of 161 patients were screened, and 140 (87%) were found to be eligible and were randomized to study interventions. There were no important differences between the experimental intervention and control group at study inclusion (*Table* 1). Overall, 127 patients (91% of randomized patients) completed treatment (experimental formula, *N* = 64; control formula, *N* = 63), were assessed at discharge and were included in the final analysis. Specifically, 13 patients did not reach the primary efficacy evaluation because of the discontinuation of the assigned nutritional intervention (product dislike). In the other patients, mean compliance to intervention was good (experimental formula, 92%; control formula, 90%). Patient disposition is illustrated in *Figure* 1. The whole randomized population and the modified ITT population were similar in terms of all the parameters taken into consideration (*Table* 1).

**Table 1:** Baseline characteristics of the study population by randomization group

Characteristic	Whole randomized population		Modified intention‐to‐treat population	
	Control formula (*N* = 70)	Experimental formula (*N* = 70)	Control formula (*N* = 63)	Experimental formula (*N* = 64)
Male gender, *N* (%)	23 (33)	29 (41)	17 (27)	26 (41)
Age (years), Mean (*SD*)	81 (5)	80 (7)	82 (5)	81 (7)
Admission diagnosis, *N* (%) Osteoarthritis Cardiovascular disease Chronic obstructive pulmonary disease Lower extremity fracture Major abdominal surgery Hypokinetic syndrome Stroke Parkinsonian syndrome	16 (22.9) 14 (20.0) 12 (17.1) 7 (10.0) 6 (8.6) 5 (7.1) 4 (5.7) 6 (8.6)	15 (21.5) 12 (17.1) 12 (17.1) 9 (12.9) 7 (10.0) 6 (8.6) 4 (5.7) 5 (7.1)	14 (22.3) 14 (22.3) 10 (15.9) 7 (11.1) 6 (9.5) 4 (6.3) 4 (6.3) 4 (6.3)	12 (18.7) 12 (18.7) 11 (17.2) 9 (14.1) 7 (10.9) 6 (9.4) 3 (4.7) 4 (6.3)
Comorbidities (*n*), Median (IQR)	6 (4–7)	6 (4–7)	6 (4–7)	6 (4–7)
Drugs (*n*), Median (IQR)	7 (5–8)	7 (5–8)	7 (5–8)	7 (5–8)
MMSE (score), Mean (*SD*)	22.1 (2.7)	21.8 (3.0)	22.0 (2.7)	21.7 (3.1)
Trail making test (s), Mean (*SD*)	44.5 (2.9)	45.9 (2.0)	44.5 (2.9)	45.9 (2.1)
Body weight (kg), Mean (*SD*)	55.5 (9.3)	54.0 (11.3)	55.5 (9.2)	54.0 (11.6)
Body mass index (kg/m^2^), Mean (*SD*)	22.1 (2.3)	21.1 (3.1)	22.1 (2.3)	21.0 (3.1)
Mini Nutritional Assessment (score), Mean (*SD*)	18.0 (2.3)	16.8 (3.1)	18.0 (2.3)	16.7 (3.1)
Appendicular muscle mass (g), Mean (*SD*)	14465 (2713)	15225.3 (3637)	14300.6 (2613)	15150.8 (3738)
Skeletal muscle mass index (kg/m^2^), Mean (*SD*)	5.78 (0.77)	5.90 (0.8)	5.73 (0.75)	5.84 (0.79)
4 meter gait speed (m/s), Mean (*SD*)	0.52 (0.10)	0.54 (0.11)	0.52 (0.09)	0.54 (0.11)
Chair stand test (s), Mean (*SD*)	28.6 (10.4)	29.1 (11.8)	29.3 (10.5)	29.1 (12.2)
Timed up and go test (s), Mean (*SD*)	24 (4.7)	23.7 (4.3)	24.0 (4.9)	23.6 (4.3)
SPPB (score), Mean (*SD*)	4.3 (0.8)	4.0 (1.2)	4.3 (0.7)	4.1 (1.2)
Handgrip strength (kg), Mean (*SD*)	19.1 (5.0)	18.2 (4.1)	18.6 (4.9)	18.2 (4.1)
Tinetti scale (score), mean (*SD*)	16.8 (3.7)	17.5 (3.7)	16.8 (3.8)	17.6 (3.6)
Barthel index (score), Mean (*SD*)	54.2 (18.9)	55.2 (18.3)	54.0 (19.3)	55.9 (18.3)
Activities of daily living (score), Mean (*SD*)	3.0 (1.4)	3.1 (0.9)	3.0 (1.4)	3.1 (0.9)
SF‐12 PCS (score), Mean (*SD*)	40.2 (11.1)	38.3 (9.6)	40.3 (11.6)	38.4 (9.8)
SF‐12 MCS (score), Mean (*SD*)	42.6 (10.4)	41.6 (10.6)	42.9 (10.9)	42 (10.7)
Rehabilitation intensity profile, Mean (*SD*) Profile ≥3, *N* (%)	3.7 (0.5) 70 (100)	3.6 (0.5) 70 (100)	3.7 (0.5) 63 (100)	3.6 (0.5) 64 (100)
Energy intake (kcal/day), Mean (*SD*)	1154.8 (160.6)	1097.9 (175.3)	1144.9 (154.2)	1095.6 (179.6)
Protein intake (g/day), Mean (*SD*)	41.8 (7.4)	42.8 (11.3)	41.5 (7.1)	42.9 (11.7)
Haemoglobin (g/dL), Mean (*SD*)	12.8 (1.4)	12.6 (1.2)	12.7 (1.5)	12.6 (1.2)
Total lymphocytes (*n*), Mean (*SD*)	2.2 (0.6)	2.3 (0.5)	2.2 (0.6)	2.3 (0.5)
Transferrin (mg/dL), Mean (*SD*)	249.5 (32.4)	249.3 (27.6)	246.4 (30.5)	248.8 (28)
Total cholesterol (mg/dL), Mean (*SD*)	179.9 (29.0)	177.2 (33.4)	178.8 (30.2)	176.2 (34.5)
Albumin (g/dL), Mean (*SD*)	3.59 (0.55)	3.6 (0.38)	3.59 (0.55)	3.6 (0.39)
Creatinine (mg/dL), Mean (*SD*)	0.84 (0.21)	0.89 (0.2)	0.83 (0.21)	0.89 (0.21)
Blood urea nitrogen (mg/dL), Mean (*SD*)	34.4 (7.0)	36.7 (6.3)	34.4 (7.2)	36.8 (6.5)
Sodium (mEq/L), Mean (*SD*)	139.7 (2.8)	139.1 (3.6)	139.8 (2.7)	139.1 (3.6)
Potassium (mEq/L), Mean (*SD*)	4.2 (0.5)	4.3 (0.6)	4.2 (0.5)	4.3 (0.5)
Calcium (mg/dL), Mean (*SD*)	9.2 (0.7)	9.2 (0.6)	9.2 (0.6)	9.2 (0.6)
Aspartate amino‐transferase (IU/L), Mean (*SD*)	19.3 (7.2)	18.1 (7.0)	19.2 (7.5)	18.1 (7.1)
Alanine amino‐transferase (IU/L), Mean (*SD*)	17.5 (9.3)	17.0 (8.1)	17.0 (9.4)	16.4 (8.1)
Blood glucose (mg/dL), Mean (*SD*)	89.8 (9.2)	86.0 (10.1)	90.2 (9.3)	86.3 (10.1)
C‐reactive protein (mg/dL), Mean (*SD*)	1.29 (1.41)	1.15 (1.38)	1.23 (1.44)	1.12 (1.4)
25‐hydroxyvitamin D (ng/mL), Mean (*SD*)	14.6 (5.6)	14.1 (8.1)	14.9 (5.7)	14.4 (8.3)

Abbreviations: IQR, interquartile range; MMSE, Mini Mental State Examination; SF‐12 MCS, 12‐item Short Form General Health Survey‐Mental Component Summary; SF‐12 PCS, 12‐item Short Form General Health Survey‐Physical Component Summary; SD, standard deviation; SPPB, Short Physical Performance Battery.

### 3.1 Primary end‐point

In the primary analysis, gait speed did not change importantly in the control group, whereas it significantly improved [+0.061 m/s/month (95%CI, 0.043 to 0.080); *P* < 0.001] in the intervention group receiving the experimental formula: mean difference, +0.062 m/s/month [95%CI, 0.043 to 0.082], *P* < 0.001. All supportive analyses yielded consistent results as the improvement persisted after adjustment for pre‐specified confounders and in the worst case scenario (*Table* 2). The crude change in gait speed at discharge was + 0.08 (SD, 0.08) m/s and + 0.00 (SD, 0.04) m/s in the experimental and control group, respectively.

**Table 2:** Analysis of the primary outcome variable [change in 4 m gait speed/month (m/s/month)] in the modified intention‐to‐treat population (patients assessed at discharge).

Analyses	Control formula Within‐group change (*N* = 63)	Experimental formula Within‐group change (*N* = 64)	Treatment effect Between‐group difference	*P* value
Primary analysis	−0.001 (−0.008 to 0.006)	0.061 (0.043 to 0.080) ^a^	0.062 (0.043 to 0.082)	<0.001
Sensitivity analyses	—	—	—	
Multivariable analysis^b^	—	—	0.042 (0.026 to 0.058)	<0.001
Worst case scenario analysis	−0.006 (−0.138 to 0.001)	0.052 (0.033 to 0.070) ^a^	0.058 (0.038 to 0.078)	<0.001

Data are provided as mean and 95%CI.

^a^ Within‐group change significant at the 5% level.

^b^ Model adjusted for sex, age, monthly change in energy intake, monthly change in creatinine, and monthly change in total cholesterol.

### 3.2 Secondary end‐points

In the experimental formula group, all the secondary end‐points addressing physical performance, functional status, and cognitive functions improved significantly vs. baseline. Specifically, improvements per month in key secondary outcome variables (physical performance) were +28% for chair‐stand test; +12.5% for TUG test; and +65% for SPPB. A substantial increase in muscle mass (AMM and SMMI) was also obtained. In the control formula group, only an improvement in SPPB (+8%) and the mental component of QoL vs. baseline was observed, while handgrip strength, the chair stand, and TUG tests worsened. Accordingly, the differences in the changes between the experimental formula group and the control formula group in terms of all the outcome parameters taken into consideration were significant, with the exception of QoL components (*Table* 3).

**Table 3:** Monthly change in secondary and exploratory physical function and nutritional outcome variables in the modified intention‐to‐treat population (patients assessed at discharge)

Endpoint	Control formula Within‐group change (*N* = 63)	Experimental formula Within‐group change (*N* = 64)	Treatment effect Between‐group difference a	P‐value
Secondary outcome variables				
MMSE (score)	−0.11 (−0.16 to −0.05) ^a^	0.46 (0.25 to 0.66) ^a^	0.57 (0.352 to 0.773)	<0.001
Trail making test (s)	−0.12 (−0.48 to 0.23)	−3.32 (−4.01 to −2.63) ^a^	−3.20 (−3.97 to −2.43)	<0.001
Barthel index (score)	0.92 (−0.15 to 1.99)	5.02 (3.77 to 6.27) ^a^	4.10 (2.47 to 5.73)	<0.001
Activities of daily living (score)	0.01 (−0.10 to 0.12)	0.67 (0.51 to 0.83) ^a^	0.66 (0.46 to 0.85)	<0.001
Tinetti scale (score)	−0.27 (−0.57 to 0.03)	2.09 (1.67 to 2.52) ^a^	2.36 (1.85 to 2.88)	<0.001
Body weight (kg)	−0.90 (−1.09 to −0.70) ^a^	1.55 (1.35 to 1.76) ^a^	2.45 (2.17 to 2.73)	<0.001
Handgrip strength (kg)	−1.47 (−2.01 to −0.92) ^a^	3.98 (3.20 to 4.75) ^a^	5.45 (4.51 to 6.38)	<0.001
SF‐12 PCS (score)	0.16 (−1.09 to 1.40)	1.47 (0.68 to 2.26) ^a^	1.31 (−0.15 to 2.77)	0.08
SF‐12 MCS (score)	1.38 (0.61 to 2.16) ^a^	1.25 (0.28 to 2.222) ^a^	−0.13 (−1.367 to 1.098)	0.82
Appendicular muscle mass (g)	−69.4 (−843.7 to 704.9)	949.8 (783.7 to 1115.8) ^a^	1019.2 (235.2 to 1803.2)	0.011
Skeletal muscle mass index (kg/m^2^)	−0.02 (−0.35 to 0.32)	0.38 (0.31 to 0.442) ^a^	0.40 (0.06 to 0.73)	0.023
SPPB (score)	0.33 (0.19 to 0.46) ^a^	2.60 (2.23 to 2.98) ^a^	2.27 (1.88 to 2.68)	<0.001
Chair stand test (s)	−4.44 (−5.85 to −3.03) ^a^	8.20 (7.05 to 9.35) ^a^	12.64 (10.84 to 14.44)	<0.001
Timed up and go test (s)	−0.76 (−1.07 to −0.44) ^a^	2.95 (2.41 to 3.49) ^a^	3.71 (3.09 to 4.33)	<0.001
Exploratory outcome variables				
Protein intake (g/day)	4.70 (3.68 to 5.71) ^a^	17.18 (15.96 to 18.41) ^a^	12.48 (10.91 to 14.06)	<0.001
Energy intake (kcal/day)	129.2 (123.6 to 134.8) ^a^	182.3 (167.9 to 196. 8) ^a^	53.1 (37.8 to 68.5)	<0.001
Mini Nutritional Assessment (score)	0.36 (0.04 to 0.68) ^a^	1.88 (1.48 to 2.27) ^a^	1.52 (1.02 to 2.02)	<0.001
C‐reactive protein (mg/dL)	0.05 (−0.11 to 0.21)	−0.38 (−0.58 to −0.19) ^a^	0.43 (0.18 to 0.68)	<0.001
25‐hydroxyvitamin D (ng/mL)	0.09 (−0.64 to 0.83)	6.70 (5.26 to 8.14) ^a^	6.61 (5.00 to 8.21)	<0.001
Total cholesterol (mg/dL)	−2.87 (−6.82 to 1.07)	1.87 (−2.70 to 6.43)	4.74 (−1.24 to 10.71)	0.12
Albumin (g/dL)	−0.14 (−0.21 to −0.07) ^a^	0.20 (0.15 to 0.25) ^a^	0.34 (0.25 to 0.43)	<0.001
Creatinine (mg/dL)	−0.01 (−0.03 to 0.01)	0.02 (0.00 to 0.05) ^a^	0.03 (0.00 to 0.06)	0.031

Data are provided as mean and 95%CI.

Abbreviations: MMSE, Mini Mental State Examination; SF‐12 MCS, 12‐item Short Form General Health Survey‐Mental Component Summary; SF‐12 PCS, 12‐item Short Form General Health Survey‐Physical Component Summary; SPPB, Short Physical Performance Battery.

^a^ Within‐group change significant at the 5% level.

More patients receiving the experimental formula went home instead of being transferred to a residential‐care facility [84.3% (*N* = 54) vs. 60.3% (*N* = 38); treatment difference, 24.0% (95%CI, 9.1 to 39.1), *P* = 0.002] and a significantly greater proportion of patients experienced a reduction in intensity of care at discharge [85.9% (*N* = 55) vs. 63.5% (*N* = 40); treatment difference, 22.5% (95%CI, 7.8 to 37.1), *P* = 0.003].

**Figure 2:**
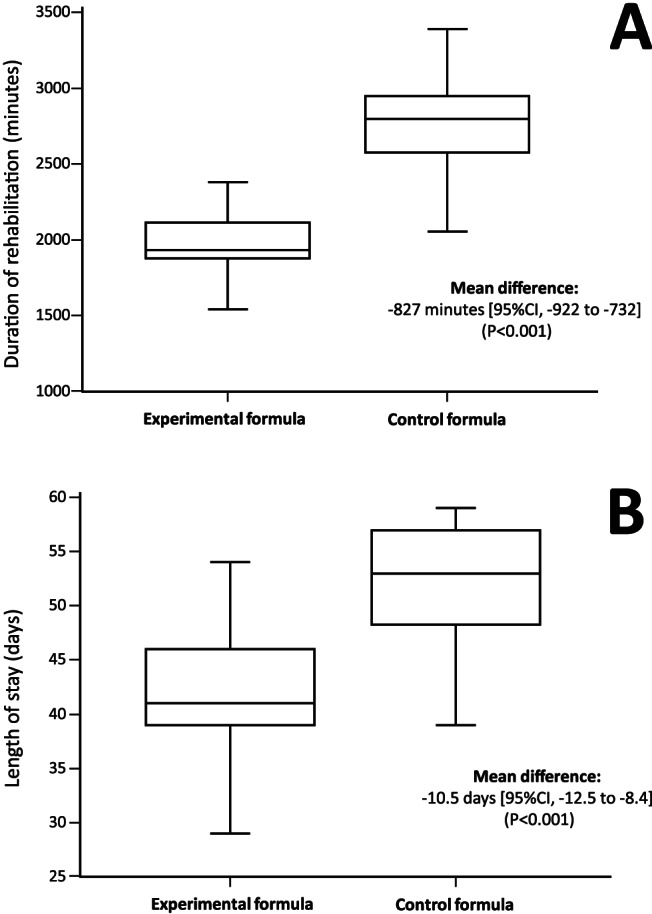
Duration of rehabilitation (A) and length of stay (B) by randomization group in the modified intention to treat population. CI, confidence interval

Finally, in respect with economic benefit surrogates, patients in the experimental intervention group required significantly less rehabilitation (*P* < 0.001) than the patients in the control formula group [mean (±SD) duration, 1986 ± 238 min vs. 2760 ± 298 min] (*Figure* 2A) and were discharged significantly earlier (mean [±SD] LOS, 41.8 ± 6.4 days vs. 52.2 ± 5.2 days) (*Figure* 2B).

### 3.3 Exploratory end‐points

Nutritional intervention resulted in an improvement in protein‐calorie intake and MNA score in both study groups, with a significantly higher effect in patients receiving the experimental formula (*P* < 0.001 for all). Furthermore, compared with control formula, patients receiving the experimental formula obtained an increase in vitamin D (*P* < 0.001), serum albumin (*P* < 0.001), and creatinine (*P* = 0.031; with no changes outside the normal range) and a reduction in C‐reactive protein (*P* < 0.001).

### 3.4 Adverse events

As reported in the study flow diagram (*Figure* 1), 13 patients discontinued the intervention because they disliked the product. In the other patients, the consumption of nutritional therapy was well tolerated (no gastro‐intestinal intolerance). No serious adverse event occurred.

## 4 Discussion

The present trial showed that, in old sarcopenic patients admitted to hospital for rehabilitation, the consumption of a muscle‐targeted whey protein‐based food for special medical purposes enriched with leucine and vitamin D improved physical performance and functional status and increased muscle mass. The intervention was also responsible for a reduction in the duration of rehabilitation and in the LOS, as well as for a higher proportion of patients discharged home, all endpoints that are associated with a substantial reduction in the costs of care. Furthermore, the study showed that in the control group, despite an increase in protein‐calorie intake, no significant improvement in clinical endpoints was achieved.

The trial, consistently with previous studies^19^, ^20^, ^21^ and in agreement with the call‐to‐action launched by the EWGSOP,^9^ sustains the importance of providing high‐quality nutritional interventions designed to support physical performance recovery in sarcopenic patients undergoing rehabilitation, as well as the need to reach protein‐calorie targets. In older adults with reduced mobility, it has been recommended that calorie and protein intake should be up to 27–30 and 1.0–1.2 g/kg/day, respectively.^41^, ^42^ Although energy intake increased significantly in both study groups (from ~20.5 to ~23.5 kcal/kg/day), it did not reach the minimum suggested target. On the other hand, mean protein intake in patients receiving the experimental formula was 1.1 g/kg/day, while it was only 0.8 g/kg/day in patients treated with the isocaloric control formula. Indeed, sarcopenia should be considered also a nutrition‐related disease and the substantial overlapping with malnutrition is now clearly codified by the core diagnostic criteria—ratified by the Global Leadership Initiative on Malnutrition (GLIM)—which include reduced muscle mass as phenotypic criterion.^43^ In this scenario, the importance of nutritional support in patients with sarcopenia is clearly emphasized as one of the expected outcomes, along with an improvement in physical performance, is muscle protein accretion.

The accepted treatment of sarcopenia consists in resistance training, optimization of protein intake, and vitamin D supplementation,^9^, ^11^ although a substantial anabolic role has been recognized for essential amino acids, particularly leucine.^14^ A recent systematic review has reported that the effectiveness of heterogeneous oral nutritional interventions—with nutritionally complete supplements or single macronutrients or micronutrient‐dense products—in combination with exercise on measures of physical functioning (e.g. gait speed and timed up and go test) in nutritionally vulnerable older adults is unconvincing despite a positive effect on muscle strength,.^44^ On the other hand, studies have reported consistent benefits from the use of muscle‐targeted nutritional supplementation.^18^, ^19^, ^20^, ^21^ However, this is the first high‐quality trial investigating the efficacy of a whey protein‐based food for special medical purposes enriched with leucine and vitamin D in combination with physical exercise on muscle function and disability as assessed through multiple physical performance outcome measures.

In a first 13 week double‐blind, placebo‐controlled trial, older sarcopenic adults living independently achieved a significant increase in total appendicular muscle mass and improvement in lower limb function (chair stand test) from this intervention even without combination with physical exercise.^18^ In a 12 week double‐blind, placebo‐controlled trial, Rondanelli et al. obtained a positive effect on muscle mass, handgrip strength, and ADL in older sarcopenic patients admitted to a geriatric rehabilitation unit for a controlled physical activity programme and receiving a similar experimental formula, while patients receiving the placebo did not improve in any of the aforementioned endpoints. However, no physical performance outcome measure was investigated.^19^ Dimori et al., using a challenge–dechallenge–rechallenge protocol in a 12 month open single‐arm study, showed that in institutionalized sarcopenic patients a multidisciplinary intervention combining a nutritional intervention and physical activity resulted in benefits in terms of muscle mass, muscle strength and physical function (gait speed and SPPB) as long as the muscle‐targeted nutritional support was administered.^20^ Finally, a recent 4 week pragmatic randomized controlled trials demonstrated that in non‐sarcopenic patients with Parkinsonian syndrome undergoing multidisciplinary intensive rehabilitation treatment adding this nutritional intervention was associated with higher improvement in lower extremity physical performance and muscle mass preservation than standard diet alone.^21^

Interestingly, the experimental intervention did not improve QoL, which was also the only subjective endpoint of our trial. The lack of effect may depend on the tool used in the assessment, which has been substantially used in and validated for the general population. The use of more specific tools, such as the Sarcopenia Quality of Life questionnaire ^45^ could have provided different responses. However, the Sarcopenia Quality of Life questionnaire has been not yet validated in the Italian language and the manuscript has been designed and approved before the validation of its English version. Another explanation may be the mild cognitive impairment characterizing the trial population. On the other hand, a positive effect on cognitive outcomes was also observed, which is consistent with available literature on the effect of improved physical fitness from exercising on cognitive health.^46^ Finally, the study not only confirmed that the intervention attenuates inflammation^19^, ^47^—a factor contributing to sarcopenia^1^, ^2^, ^4^, ^5^—but also clearly demonstrated for the first time that a high‐quality nutritional support in patients undergoing rehabilitation enables the achievement of improved physical function with substantial reduction in the costs of care. Although this was a secondary endpoint and a formal cost‐effectiveness analysis was not conducted, we could grossly estimate that a small investment in nutritional supplementation results in shorter LOS and physical therapy. This is clearly an important issue for resource allocators, taking into account the adverse health outcomes that incur heavy burden for patients and healthcare systems^9^, ^10^ and the age‐ and gender‐specific prevalence projections, which indicate that in 2045 the number of individuals affected in Europe will rise from 10–20 million to 20–30 million.^48^

In respect to the discussion of the relevance of present results, it should be highlighted that the scientific community is currently working on the selection of the primary endpoints and the evaluation of the appropriate thresholds to be used in trials in sarcopenia,^49^, ^50^ and a major problem is the need to define clinically meaningful changes in almost all relevant outcome variables. Although all changes in muscle function and physical performance endpoints were found to be statistically significant in our trial, they could not appear clinically meaningful due also to the normalization for the LOS. However, specifically for the primary outcome variable on which the study was sized, the crude mean difference was 0.08 m/s. In previous disease‐specific settings (old adults with mobility disabilities, subacute stroke survivors, and hip fracture recovery)^51^, ^52^, ^53^ improvements near 0.10 m/s have been described to be substantial, although baseline conditions likely affect this estimate.^54^ Interestingly, present trial results are also consistent with the effect of the same nutritional formula provided along with a multidisciplinary intensive rehabilitation programme in patients with Parkinsonian syndromes (+0.07 m/s).^21^ Nonetheless, change in SPPB—a composite measure of physical performance—has been considered to be relevant when around 1.5 points. In our study, the crude change was +3.4 (SD, 1.7) points and + 0.5 (SD, 1.1) points in the experimental and control group, respectively (mean difference, +2.9 points).

As additional limitations, we recognize that the study has been conducted at a single site and has addressed only the short‐term efficacy of a multidisciplinary intervention. We do not know how effective this intervention could be in the long‐term. A previous report has suggested that physical exercise alone is not enough to maintain the improvement in physical performance and that both interventions should be continued.^20^ Nonetheless, in our trial, all patients followed an individualized physical activity rehabilitation programme, but benefits were seen only in the group receiving the muscle‐targeted nutritional formula, indicating that a combined approach is mandatory for the treatment of sarcopenia.

A major strength of the study is the measurement of muscle function and physical performance through a large number of approaches. A recent position paper by European Society for Clinical and Economic Aspects of Osteoporosis and Osteoarthritis has highlighted that different methods are available. Although the choice of the tool should consider different aspects (purpose of the assessment, patient characteristics, psychometric properties, applicability in clinical settings, and prognostic reliability) and the use of some of them is advised (handgrip strength, 4 m gait speed test and SPPB test) based on the more robust responsiveness, we do believe that the extensive evaluation conducted in our trial further emphasizes the value of the intervention..^55^ We also consider as points of strength the conduction in a real‐life geriatrics rehabilitation setting and the adoption of less stringent exclusion criteria. Although some restrictions were inevitable, they do not seem to affect data generalizability significantly as less than 10% of patients screened were not eligible. The exclusion of patients with moderate‐severe cognitive impairment does not seem to be a major limitation. Furthermore, we included patients with a diagnosis of sarcopenia based on the old operational EWGSOP criteria.^8^ Although the study has been designed and completed before the release of the updated EWGSOP2 criteria^9^ and no protocol amendment can be considered at this time, we can report that the study is still up to date as all patients were suffering from severe sarcopenia (characterized by low muscle mass, low muscle strength, and reduced physical performance). This is reasonably the profile of the patients admitted to this setting.

In conclusion, in old adults with sarcopenia admitted to hospital for rehabilitation the consumption of a muscle‐targeted whey protein‐based nutritional formula enriched with leucine and vitamin D improved physical performance and function, as well as muscle mass, and reduced the intensity and costs of care. Confirmatory trials addressing the value of a muscle‐targeted nutritional formula in combination with exercise on the key features of sarcopenia are needed and benefits should be also explored in patients with preserved physical performance.

## Conflict of interest

All authors have completed the Unified Competing Interest form (available on request from the corresponding author) and declare: no support from any organisation for the submitted work.

Dr Cereda reports the following conflicts of interest (not for the present study):

- Consulting or Advisory Role: Nutricia S. p. A., Akern S.r.l., Wunder Sa.Bi. s.r.l., Fondazione Grigioni per il Morbo di Parkinson.
- Speaker's Honoraria: Nutricia S. p. A., Nestlè Health Science.

Dr Klersy is a statistical consultant with Nutricia S. p. A. (inclusive of the present work). All the other authors declare no financial relationships with any organisations that might have an interest in the submitted work in the previous three years. All authors declare no other relationships or activities that could appear to have influenced the submitted work.

## Data sharing statement

We provide qualified researchers access to study protocol and statistical analysis plan (available from the corresponding author). Individual participant data will not be shared due to ethical reasons.

## Statement of authorship

Prof Dr Rondanelli had full access to all of the data in the study and takes responsibility for the integrity of the data and the accuracy of the data analysis. Dr Emanuele Cereda affirms that the manuscript is an honest, accurate, and transparent account of the study being reported; that no important aspects of the study have been omitted; and that any discrepancies from the study as planned have been explained. All the authors significantly contributed to the work and approve the manuscript for submission. Particularly, contributions were as follows: Cereda, Perna, and Rondanelli performed the study concept and design. Faliva, Gasparri, Infantino, Nichetti, Peroni, Rondanelli, and Spadaccini was tasked for the acquisition of data. Caccialanza, Cereda, Iannello, Klersy, Perna, and Rondanelli analysed and interpreted the data. Drafting of the manuscript was performed by Cereda and Rondanelli. Critical revision of the manuscript for important intellectual content was performed by Caccialanza, Cereda, Iannello, Perna, and Rondanelli. Klersy performed the statistical analysis whereas Rondanelli obtained the funding. Administrative, technical, or material support was given by Perna and Rondanelli. Lastly, Perna and Rondanelli supervised the study.

## Supporting information



Data S1. Supporting Information
