# Supplementary material for: Improving rehabilitation in sarcopenia: a randomized‐controlled trial utilizing a muscle‐targeted food for special medical purposes
Source: J Cachexia Sarcopenia Muscle. 2020 Sep 22;11(6):1535–47. doi: 10.1002/jcsm.12532 (PMC7749532; doi:10.1002/jcsm.12532)
Supplement: Supplementary file 1 — Data S1. Supporting Information [file JCSM-11-1535-s001.doc]

Study protocol

***“*Improving Rehabilitation In Sarcopenia (IRIS) by muscle-targeted food for special medical purposes: a randomized, double-blind, controlled trial*”***

## ClinicalTrials.gov Identifier: NCT03120026

Principal Investigator:

Prof. Mariangela Rondanelli M.D., PhD

Department of Public Health, Experimental and Forensic Medicine, Unit of Human and Clinical Nutrition, University of Pavia, Italy

Via Emilia 12, Pavia

tel. 0382-381749

E-mail: [mariangela.rondanelli@unipv.it](mailto:mariangela.rondanelli@unipv.it)

Corresponding Author:

Emanuele Cereda MD, PhD

Clinical Nutrition and Dietetics Unit, Fondazione IRCCS Policlinico San Matteo

Viale Golgi 19, 27100 Pavia, Italy.

Tel.: +39 0382 501615 ; Fax: + 39 0382 502801

E-mail: [e.cereda@smatteo.pv.it](mailto:e.cereda@smatteo.pv.it)

| **Revisions** | **Date** | **Who** | **Ethics Committee approval** |
| --- | --- | --- | --- |
| 1 (original) | February 2017 | M. Rondanelli | March 2017 |

**TABLE OF CONTENTS**

1. Introduction 4

2. Objective & study enpoints 5

2.1 Primary endpoint 5

2.2 Secondary endopoints 5

2.3 Other pre-specified study endpoints 5

3. Study design 6

4. Participants 6

4.1 Inclusion criteria 6

4.2 Exclusion criteria 6

5. Intervention 7

5.1 Physical intervention 7

5.2 Nutritional intervention 7

6. Methods 8

6.1 Assessments 9

6.2 Assessment of the primary endpoint (efficacy) 11

6.3 Assessment of safety 11

7. Data management 11

7.1 Data collection 11

7.2 Database management and quality control 11

8. Statistics and sample size 12

8.1 Sample size 12

8.2 Statistical Analysis 12

9. Reference list 13

# **Introduction**

Age-related and disease-related loss of muscle mass and strength induces important negative pathophysiological changes in the elderly, such as reduction in the ability to maintain balance, changes in glucose homeostasis, thermoregulation and bone nutrition, and reduced basal metabolic rate/energy production. These consequences contribute considerably to the progression of loss of self-sufficiency on account of the reduction in both dynamic and static muscle strength, increase in morbidity, and susceptibility to multiple disorders; overall, they contribute to a general condition called sarcopenia. A number of factors may contribute to its onset. Sudden and important muscle mass loss is a common occurrence following a period of immobility or reduction in mobility, such as admission to hospital, and only three days of bed rest can result in the loss of >1 kg of muscle mass in elderly patients. Nonetheless, sarcopenia is a disease that frequently occurs in the community where a sedentary lifestyle and inadequate dietary intake of proteins and specific micronutrients (e.g. vitamin D) can trigger and accelerate its progression.

Taking into account the time trends in life expectancy, the treatment of this disease is a key issue with important pharmaco-economic implications. Interventions should modulate anabolic and catabolic pathways within the muscle but, despite a substantial increase in the understanding of the molecular basis, approved agents are limited and not free of side effects. At present, the basic treatment of sarcopenia consists in targeting muscle mass and function through physical exercise (resistance training), optimization of protein intake and vitamin D supplementation.

In respect to nutritional intervention, particularly to optimize protein intake in advanced age, attention should be paid not only to the amount of proteins provided but also to their quality. Whey proteins have proved to enable a greater anabolic stimulation due to their faster digestion – resulting in a more rapid increase in plasma amino acid levels – and high content in essential amino acid. Among essential amino acids, leucine has been found to stimulate anabolism independently. It has been recommended that a per-meal anabolic threshold of essential amino acids, particularly in terms of leucine (2.5–2.8 g/meal), should be achieved at least twice daily. Besides, vitamin D supplementation was found to result in improved muscle strength, particularly in old adults with low serum levels of the vitamin, and to promote muscle anabolism also through a positive interaction among all these nutrients.

Recent studies have demonstrated that the use of a muscle-targeted food for special medical purposes (a mixture of whey proteins enriched with essential amino acids, especially leucine, vitamin D and calcium) improves muscle mass and strength. However, although clinical trials in sarcopenia should address the recovery of physical functioning, the efficacy of the muscle-targeted food for special medical purposes on physical performance has never been evaluated in combination with physical exercise in a high-quality trial. Furthermore, there are no data on the economic benefits deriving from nutritional support in in-patient rehabilitation facilities.

# **Objective**

The aim of the study is to investigate the efficacy of a muscle-targeted nutritional supplementation (a mixture of serum proteins enriched with essential amino acids, especially leucine, vitamin D and calcium) on physical performance, functional and muscle mass recovery in older patients with sarcopenia admitted to an in-patient rehabilitation facility.

***2.1 Primary endpoint***

The primary endpoint will be the mean change in gait speed per month (m/s/month) during the 4-meter walking test.

***2.2 Secondary endpoints***

The following key secondary endpoints (change per month in physical performance outcome measures) will be investigated:

- chair-stand test
- Timed up and go (TUG) test
- Short Physical Performance Battery (SPPB)

The following secondary endpoints will be also addressed:

- change per month in handgrip strength
- change per month in Tinetti scale
- change per month in Barthel Index
- change per month in activities of daily living (ADL) score
- change per month in body weight
- change per month in appendicular muscle mass (AMM)
- change per month in skeletal muscle mass index (SMMI)
- change per month in cognitive status (Trail making test and Mini Mental State Examination [MMSE])
- change per month in quality of life (Short-Form 12-Item Health Survey [SF-12])
- the proportion of patients who improved their rehabilitation intensity profile
- the modality of discharge (home vs. institution)
- the overall economic benefits (using length of stay [LOS] and total duration of rehabilitation [minutes] as surrogate measures)
- safety of the intervention

***2.3 Other pre-specified study endpoints***

As explorative endpoints, we will also consider changes (per month) in: protein and energy intake, Mini Nutritional Assessment (MNA®) score, C-reactive protein, vitamin D, total cholesterol, albumin and creatinine.

# **Study design**

A single-site, interventional, randomized (1:1), parallel-group, double-blind, controlled, 8-week clinical trial.

Expected study duration: 24 months.

# **Participants**

Consecutive, old adults, candidates for in-patient rehabilitation, who were found to have sarcopenia.

## *4.1 Inclusion criteria*

- age ≥65 years
- admission for physical rehabilitation
- sarcopenia (according to European Working Group on Sarcopenia in Older People [EWGSOP] criteria in terms of outcome of body composition by bioimpedance analysis [skeletal muscle mass/body weight × 100, ≤37% in men and ≤28% in women], handgrip strength and gait speed)
- Mini Mental State Examination (MMSE) ≥18
- Written informed consent (patient or legal guardian)

## *4.2 Exclusion criteria*

- Any malignant disease during the last five years
- Known kidney failure (previous glomerular filtration rate <30 ml/min);
- Known liver failure (Child-Pugh class B or C)
- Known psychiatric disorder
- Endocrine disorders associated with disorders of calcium metabolism (except osteoporosis)
- Indications related to the study product:
  - - More than 10 µg (400 IU) of daily Vitamin D intake from medical sources
    - More than 500 mg of daily calcium intake from medical sources.
    - Adherence to a high energy or high protein diet up or use of protein containing or amino acid containing nutritional supplements up to three months before starting the study.
- Known allergy to milk, milk products or other components of the proposed interventions
- Indication to or ongoing artificial nutrition support
- Inclusion in other nutrition intervention trials
- Investigator's uncertainty about the willingness or ability of the subject to comply with the protocol requirements
- Refusal

# **Intervention**

## *5.1 Physical intervention*

An individualized, moderate-level (Borg Rate of Perceived Exertion scale score of 12-14) physical fitness and muscle mass promoting program will be set up for all in-patients. Trained staff will supervise all exercise sessions, monitoring the individual exercise ability of each patient and adjusting the intensity level, as appropriate. The intervention will consist of exercise sessions daily, 5 times/week. The initial duration of each session will be 20 minutes and it could be increased progressively, along with the intensity of the exercises, up to 30 minutes. All sessions will include the following:

- 5-minute warm-up

- 5-to-10-minute progressive sequence from seated to standing muscle-strengthening exercises: toe raises, heel raises, knee lifts, knee extensions in the seated position; hip flexions and lateral leg raises standing next to a chair used for stability; ankle-weight bearing exercises (seated knee flexion and extension, standing knee flexion and extension), with weights ranging from 0.5 to 1.5 kg as appropriate (in accordance with each participant’s strength as the resistance progressively increased); leg extensions and hip flexions using resistance bands. Upper-body exercises will be also performed and included double-arm pull downs and biceps curls. Patients will be asked to perform up to 8 repetitions, as appropriate

- 5-to-10-minute balance and gait exercises: one-leg stands, tandem stands, multidirectional weight shifts, tandem walk, as well as practicing proper gait mechanics focusing on balance maintenance and increasing stride length, while changing direction and/or gait pattern

- 5-minute cool-down.

The minimum duration of the physical intervention program will be 4 weeks and it could be prolonged up to 8 weeks according to the results obtained. Specifically, the decision to finish the rehabilitation and to discharge the patient will be taken by a multidisciplinary team (geriatrician, physiatrist, physiotherapist and nurse) once the duration of each exercise session is stabilized to 30 minutes and no increase in intensity could be considered for 5 consecutive days.

***5.2 Nutritional intervention***

An individualized dietary program will be drawn up for each patient, taking nutritional and mastication issues, as well as any swallowing issues into consideration. In addition to hospital diet, subjects will be randomly allocated to receive twice daily:

a) Experimental formula: a whey protein-based food for special medical purposes enriched with leucine and vitamin D (Fortifit®, Nutricia). Each serving consists of 40 g of powder (vanilla or strawberry flavor), providing 150 kilocalories and containing 20 g of whey proteins, 2.8 g of leucine, 9 g of carbohydrates, 3 g of fat, 800 IU of vitamin D, and a mixture of vitamins, minerals (calcium, 500 mg), and fibers [17-20].

b) Control formula: an isocaloric formula consisting of 40 g of a flavored (vanilla or strawberry) powder containing maltodextrins.

The intervention formula will be reconstituted with 100-150 mL water and administered at breakfast and in the afternoon. In the event of dysphagia to liquids, the density of the reconstituted formula will be increased, as appropriate.

The actual supplement and control formula will be given in identical containers devoid of any labelling for at least 4 weeks (minimum duration of the rehabilitation) up to 8 weeks (maximum duration of the rehabilitation). Compliance with intake of nutritional interventions will be monitored by recording the number of servings consumed every day in a diary.

1. **Methods**

The assessments and the procedures to be performed throughout the study are summarized in the following table:

| ASSESSMENTS/PROCEDURES | BASELINE | END OF STUDY |
| --- | --- | --- |
| Inclusion/exclusion criteria | X |  |
| Informed consent | X |  |
| Medical history & demographics | X |  |
| Randomization | X |  |
| Anthropometry | X | X |
| Protein-calorie intake | X | X |
| Mini Nutritional Assessment | X | X |
| Physical performance | X | X |
| Physical function | X | X |
| Muscle strength (handgrip) | X | X |
| Muscle mass | X | X |
| Cognitive function | X | X |
| Biochemistry | X | X |
| Quality of life | X | X |
| Complexity of assistance needs | X | X |
| Compliance to nutritional intervention |  | X |
| Safety data |  | X |
| Modality of discharge |  | X |
| Total duration of rehabilitation |  | X |
| Length of stay |  | X |

## *6.1 Assessments*

The following assessments will be performed throughout the study as indicated above:

*Medical History & Demographics.* Information will be collected on age, gender, main admission diagnosis, number of comorbidities and medications.

*Nutritional Assessment:* body weight (to the nearest 0.1 kg) and height (to the nearest 0.5 cm) will be measured according to standard procedures and body mass index (BMI) will be derived accordingly (*Anthropometry*). A trained dietitian will be responsible for the evaluation of calorie and protein intake. At study inclusion a 24-h dietary recall (with the aid of the caregiver) will be performed with the help of a food atlas, while at the end of study a calibrated dietetic spring scale will be used to weigh all foods served and returned on consecutive days. A computer program (DR3 v3.1.0; Sintesi Informatica Srl, Milano, Italy) will be used to estimate the energy and the macronutrient content of food consumed (*Protein-calorie intake*), including nutritional supplementation. Finally, nutritional status will be rated by means of the *Mini Nutritional Assessment* (MNA®), a brief questionnaire based on an anthropometric assessment (BMI and weight loss), a general assessment (lifestyle, medication, and mobility), and a dietary assessment (number of meals, food and fluid intake, self-assessment of autonomy of eating, and self-perception of health and nutrition).

*Muscle mass (body composition)*: in the screening phase, the presence of low skeletal muscle mass will be diagnosed [(skeletal muscle mass/body weight × 100) ≤37% in men and ≤28% in women] using bioelectric impedance analysis (BIA 101; Akern s.r.l., Florence, Italy). Then, appendicular muscle mass (AMM) and total body skeletal muscle mass (for the calculation of skeletal muscle mass index [SMMI]) will be evaluated using Dual-energy X-ray absorptiometry (DXA; Lunar Prodigy, GE Medical Systems).

*Evaluation of physical performance*: it will comprise multiple tests. Gait speed will be evaluated by the 4-meter walking test. Lower body leg strength and endurance will be investigated through the chair-stand test (time required to rise 5 consecutive times from a chair without arm rests). Composite evaluation of mobility, balance, walking ability, and fall risk will be performed using the Timed up and go (TUG) test, which assesses the time taken to rise from an arm chair, walk 3 meters, turn, walk back, and sit down again. Finally, we will consider the Short Physical Performance Battery (SPPB) which consists of 3 components: gait speed, chair-stand test, TUG, and balance (3 different tests assessing ability to stand with the feet together in the side-by-side, semi-tandem, and tandem positions). Accordingly, each component is scored from 0 (not possible) to 4 (best performance); the scores add up to a total score ranging from 0 to 12.

*Functional status evaluation*: it will include muscle strength measured as handgrip strength (according to standard procedures by a hydraulic hand dynamometer [Jamar 5030J1; Sammons Preston Rolyan; Bolingbrook, Canada]; accuracy 0.6 N]), the Barthel Index (BI; covering all the aspects of self-care independence in daily living activities including transfer, walking, stairs, toilet use, dressing, feeding, bladder, bowel, grooming, bathing; score range, 0 [completely dependent] - 100 [complete self-sufficiency]), activities of daily living (ADL) score, and the Tinetti scale which measures characteristics associated with falls, assessing balance (14 items; 24 points) and gait (10 items; 16 points) for a total score up to 40 (the higher the score, the better the performance).

*Evaluation of cognitive functions*: it will include the MMSE (a 30-point questionnaire used to measure cognitive impairment assessing functions including registration, attention and calculation, recall, language, ability to follow simple commands and orientation) and the Trail making test a neuropsychological test of visual attention and task switching, providing information about visual search speed, scanning, speed of processing, mental flexibility, as well as executive functioning (the score is obtained as the number of seconds needed to complete the test).

*Quality of life assessment*: participants will be tested with the Short-Form 12-Item Health Survey (SF-12), a short, generic health‐status measure reproducing the 2 summary scores of the SF‐36 - the physical component summary (PCS) score and the mental component summary (MCS) score – by addressing 8 health domains (physical functioning, role physical, bodily pain, general health, vitality, social functioning, role emotional, and mental health.

*Biochemical assessment*: venous blood samples will be drawn after an over-night fast and used for the evaluation of routine parameters (total blood count, glucose, transaminases, albumin, creatinine, blood urea nitrogen, serum electrolytes, transferrin, total cholesterol), as well as C-reactive protein and 25-hydroxyvitamin D (25(OH)D) levels.

*Assessment of complexity of assistance needs*: the rehabilitation profile system will be used to rate the complexity of assistance needed by the patient. Profiles are defined according to the assessment of 4 kinds of intervention and their interaction (general assistance, functional reactivation and recovery, medical support and social support). Patients entering an intermediate rehabilitation program as in-patients can be assigned any one of 5 different profiles:

- Profile 1: low assistance and medical needs; patient requires mainly general assistance;
- Profile 2: intermediate general assistance needs, but low medical needs; patient requires mainly general assistance, as well as functional reactivation and recovery;
- Profile 3: high general assistance needs and intermediate medical needs; patient requires mainly general assistance, functional reactivation and recovery, as well as intermediate medical support;
- Profile 4: high assistance and medical needs; patient requires general assistance, functional reactivation and recovery, as well as medical support on account of important concomitant diseases;
- Profile 4B DEMENTIA: high assistance and medical needs. Patient suffers from dementia and therefore requires a lot of general assistance, functional reactivation and recovery, as well as medical and social support provided by highly trained professionals.

## *6.2 Assessment of the primary endpoint (efficacy)*

The primary endpoint will be the mean change in gait speed at discharge. Particularly, expecting a treatment effect also on the duration of hospital stay, the change over time will be normalized by the duration of observation in months. Gait speed will be evaluated by the 4-meter walking test, asking the patients to walk at their usual pace. Patients will be allowed to use an assistive device, if needed. Specifically, the patient will be asked to walk down a hallway through a 1-metre zone for acceleration, a central 4- metre “testing” zone, and a 1-metre zone for deceleration (the patient should not start to slow down before the 4-metre mark), starting and stopping the timer with the first footfall after the 0-metre line and the 4-metre line, respectively. The best time of two attempts will be taken into account.

## *6.3 Assessment of safety*

Based on previous trials investigating the use of this food for special medical purposes, the proposed intervention is considered to be safe. Patients will be actively monitored for the occurrence of any potential gastrointestinal side effect associated with the consumption of the nutritional intervention formula (common adverse events). The occurrence of any unexpected serious adverse event will also be recorded.

# **Data Management**

## *7.1. Data collection*

A single person (registered dietitian) will be responsible for data input. The patients will be identified by a registration number and will remain anonymous. Data will be progressively (after each evaluation) collected in a locked electronic database (Microsoft® Office EXCEL). At the end of the study, data will be verified for accuracy and completeness.

## *7.2. Database management and quality control*

After the last evaluation of the last patient, the database will be checked for data quality and plausibility. This will be completed with the treatment codes (*Treatment A* or *Treatment B*), which will be masked during the statistical analysis (database closed).

# **Statistics and sample size**

***8.1 Sample size***

In the absence of preliminary data to estimate the expected treatment difference, the sample size was set at 128 patients reaching the evaluation of the primary endpoint (64 per arm) to achieve a statistical power of 80% (type I error 5% using a two-tailed test) to detect a clinically meaningful difference (mean treatment difference/standard deviation [effect size]=0.5). Allowing for a 10% drop-out rate in each arm, it was decided to randomize 140 patients (70 per treatment arm).

***8.2 Statistical analysis***

The efficacy analysis population will include the patients reaching the primary endpoint evaluation and it will be performed according to the intention to treat principle.

All patients consuming at least one serving of nutritional formula will be included in the safety analysis.

Descriptive statistics: Mean and standard deviation, range, median and quartiles will be calculated for continuous variables. Frequencies and percentages will be calculated for categorical variables.

Analysis of study endpoints: Expecting a treatment effect also on the LOS, changes over time in both primary and secondary continuous outcome variables will be normalized by the duration of observation in months. Changes will be calculated so that an improvement would result in a positive value in favor of the experimental formula (as either final - initial value or initial - final values).

*Primary endpoint* - The change in gait speed will be compared between groups with a generalized linear regression model using Huber-White robust standard errors to account for variance inhomogeneity. Then a series of supportive analyses of the primary endpoint will be performed. First, we will conduct a multivariable model, to adjust for potential confounders regardless of differences in baseline features, including gender, age and monthly change in energy intake, creatinine and in total cholesterol. Second, a conservative sensitivity analysis of the primary endpoint using the worst possible outcome of the study for patients dropping out will be performed.

*Secondary endpoints* - Group comparison will be performed for all secondary endpoints on a continuous scale using an unadjusted generalized linear regression model. For both primary and secondary outcome variables the mean change within groups will be also assessed. For secondary endpoints on a binomial scale, a generalized linear model for the binomial family will be used. For all endpoints the treatment effect (mean or frequency difference) and 95% confidence interval (CI) will be reported.

All statistical analyses will be performed using the software STATA 15.0 statistical software (Stata Corporation, College Station, TX).

Dropout

The following causes of dropout will be considered:

- Death

- Lost to follow-up (due to hospitalization or transfer to another setting of care)

- Withdrawal

***Reference list***

Abellan van Kan G, Rolland Y, Andrieu S, Bauer J, Beauchet O, Bonnefoy M, Cesari M, Donini LM, Gillette Guyonnet S, Inzitari M, Nourhashemi F, Onder G, Ritz P, Salva A, Visser M, Vellas B. Gait speed at usual pace as a predictor of adverse outcomes in community-dwelling older people an International Academy on Nutrition and Aging (IANA) Task Force. J Nutr Health Aging. 2009 Dec;13(10):881-9.

Anker SD, Morley JE, von Haehling S. Welcome to the ICD-10 code for sarcopenia. J Cachexia Sarcopenia Muscle. 2016 Dec;7(5):512-514.

Bauer J, Biolo G, Cederholm T, Cesari M, Cruz-Jentoft AJ, Morley JE, Phillips S, Sieber C, Stehle P, Teta D, Visvanathan R, Volpi E, Boirie Y. Evidence-based recommendations for optimal dietary protein intake in older people: a position paper from the PROT-AGE Study Group. J Am Med Dir Assoc. 2013 Aug;14(8):542-59.

Bauer JM, Verlaan S, Bautmans I, Brandt K, Donini LM, Maggio M, McMurdo ME, Mets T, Seal C, Wijers SL, Ceda GP, De Vito G, Donders G, Drey M, Greig C, Holmbäck U, Narici M, McPhee J, Poggiogalle E, Power D, Scafoglieri A, Schultz R, Sieber CC, Cederholm T. Effects of a vitamin D and leucine-enriched whey protein nutritional supplement on measures of sarcopenia in older adults, the PROVIDE study: a randomized, double-blind, placebo-controlled trial. J Am Med Dir Assoc. 2015 Sep 1;16(9):740-7.

Beaudart C, Buckinx F, Rabenda V, Gillain S, Cavalier E, Slomian J, Petermans J, Reginster JY, Bruyère O. The effects of vitamin D on skeletal muscle strength, muscle mass, and muscle power: a systematic review and meta-analysis of randomized controlled trials. J Clin Endocrinol Metab. 2014 Nov;99(11):4336-45.

Borack MS, Volpi E. Efficacy and safety of leucine supplementation in the elderly. J Nutr. 2016 Dec;146(12):2625S-2629S.

Borg GA. Psychophysical bases of perceived exertion. Med Sci Sports Exerc 1982;14(5):377-81

Cavaliere, B. Metodo di determinazione degli Indici di Complessità Assistenziale (ICA): dieci anni di sviluppo e sperimentazione. AICM Journal - Giornale Italiano di Case Management. 2013;2(1):6-15

Cereda E. Mini nutritional assessment. Curr Opin Clin Nutr Metab Care. 2012 Jan;15(1):29-41.

Cohen J. A Power primer. Psychol Bull. 1992 Jul;112(1):155-9.

Cruz-Jentoft AJ, Baeyens JP, Bauer JM, Boirie Y, Cederholm T, Landi F, Martin FC, Michel JP, Rolland Y, Schneider SM, Topinková E, Vandewoude M, Zamboni M; European Working Group on Sarcopenia in Older People. Sarcopenia: European consensus on definition and diagnosis: Report of the European Working Group on Sarcopenia in Older People. Age Ageing. 2010 Jul;39(4):412-23.

Dangin M, Guillet C, Garcia-Rodenas C, Gachon P, Bouteloup-Demange C, Reiffers-Magnani K, Fauquant J, Ballèvre O, Beaufrère B. The rate of protein digestion affects protein gain differently during aging in humans. J Physiol. 2003 Jun 1;549(Pt 2):635-44.

Dhillon RJ, Hasni S. Pathogenesis and Management of Sarcopenia. Clin Geriatr Med. 2017 Feb;33(1):17-26.

Guralnik JM, Simonsick EM, Ferrucci L, Glynn RJ, Berkman LF, Blazer DG, Scherr PA, Wallace RB. A short physical performance battery assessing lower extremity function: association with self-reported disability and prediction of mortality and nursing home admission. J Gerontol. 1994 Mar;49(2):M85-94.

Hardy R, Cooper R, Shah I, Harridge S, Guralnik J, Kuh D. Is chair rise performance a useful measure of leg power? Aging Clin Exp Res. 2010 Oct-Dec;22(5-6):412-8.

Istituto Scotti Bassani (1989) Atlante Ragionato di Alimentazione. Istituto Scotti Bassani per la ricerca e l'informazione scientifica e nutrizionale, Milano

Janssen I, Baumgartner RN, Ross R, Rosenberg IH, Roubenoff R. Skeletal muscle cutpoints associated with elevated physical disability risk in older men and women. Am J Epidemiol. 2004 Feb 15;159(4):413-21

Kalyani RR, Corriere M, Ferrucci L. Age-related and disease-related muscle loss: the effect of diabetes, obesity, and other diseases. Lancet Diabetes Endocrinol. 2014 Oct;2(10):819-29.

Lawton MP, Brody EM. Assessment of older people: self-maintaining and instrumental activities of daily living. Gerontologist. 1969 Autumn;9(3):179-86.

Magni E, Binetti G, Bianchetti A, Rozzini R, Trabucchi M. Mini-Mental State Examination: a normative study in Italian elderly population. Eur J Neurol. 1996 May;3(3):198-202.

Mahoney FI, Barthel D. Functional evaluation: the Barthel Index. Md State Med J. 1965 Feb;14:61-5.

Norman G, Monteiro S, Salama S. Sample size calculations: should the emperor’s cloche be off the peg or made to measure? BMJ 2012;345:e5278.

Podsiadlo D, Richardson S. "The timed "Up & Go": a test of basic functional mobility for frail elderly persons." J Am Geriatr Soc 1991;39(2):142-148.

Rondanelli M, Klersy C, Terracol G, Talluri J, Maugeri R, Guido D, Faliva MA, Solerte BS, Fioravanti M, Lukaski H, Perna S. Whey protein, amino acids, and vitamin D supplementation with physical activity increases fat-free mass and strength, functionality, and quality of life and decreases inflammation in sarcopenic elderly. Am J Clin Nutr. 2016 Mar;103(3):830-40.

Rudrappa SS, Wilkinson DJ, Greenhaff PL, Smith K, Idris I, Atherton PJ. Human Skeletal Muscle Disuse Atrophy: Effects on Muscle Protein Synthesis, Breakdown, and Insulin Resistance-A Qualitative Review. Front Physiol. 2016 Aug;7:361

Salles J, Chanet A, Giraudet C, Patrac V, Pierre P, Jourdan M, Luiking YC, Verlaan S, Migné C, Boirie Y, Walrand S. 1,25(OH)2-vitamin D3 enhances the stimulating effect of leucine and insulin on protein synthesis rate through Akt/PKB and mTOR mediated pathways in murine C2C12 skeletal myotubes. Mol Nutr Food Res. 2013 Dec;57(12):2137-46.

Tinetti ME. Performance-oriented assessment of mobility problems in elderly patients. J Am Geriatr Soc. 1986 Feb;34(2):119-26.

Tombaugh TN. Trail Making Test A and B: normative data stratified by age and education. Arch Clin Neuropsychol. Mar;19(2):203-14.

Vellas B, Fielding R, Bhasin S, Cerreta F, Goodpaster B, Guralnik JM, Kritchevsky S, Legrand V, Forkin C, Magaziner J, Morley JE, Rodriguez-Manas L, Roubenoff R, Studenski S, Villareal DT, Cesari M; International Conference on Frailtyand Sarcopenia Research Task Force. Sarcopenia Trials in Specific Diseases: Report by the International Conference on Frailty and Sarcopenia Research Task Force. J Frailty Aging. 2016;5(4):194-200.

Vitale G, Cesari M, Mari D. Aging of the endocrine system and its potential impact on sarcopenia. Eur J Intern Med. 2016 Nov;35:10-15.

Wall BT, Dirks ML, van Loon LJ. Skeletal muscle atrophy during short-term disuse: implications for age-related sarcopenia. Ageing Res Rev. 2013 Sep;12(4):898-906.

Ware J Jr, Kosinski M, Keller SD. A 12‐item Short‐Form Health Survey: construction of scales and preliminary tests of reliability and validity. Med Care 1996 Mar;34(3):220-33.

World Health Organization. Physical status: the use and interpretation of anthropometry. Report of a WHO Expert Committee. World Health Organ Tech Rep Ser 1995;854:1-452.
